# Pathfinding: a neurodynamical account of intuition

**DOI:** 10.1038/s42003-025-08612-9

**Published:** 2025-08-13

**Authors:** Steven Kotler, Michael Mannino, Karl Friston, Gyorgy Buzsáki, J. A. Scott Kelso, Guillaume Dumas

**Affiliations:** 1 Flow Research Collective, Gardnerville, NV USA; 2 Artificial Intelligence Center, Miami Dade College, Miami, FL USA; 3 University College London, Queen Square Institute of Neurology, London, UK; 4 Department of Neuroscience and Physiology, Grossman School of Medicine, New York University, New York City, NY USA; 5 Center for Complex Systems and Brain Sciences, Florida Atlantic University, Boca Raton, FL USA; 6 Intelligent Systems Research Centre, Ulster University, Derry~Londonderry, Northern Irealand UK; 7 Bath Institute for the Augmented Human, University of Bath, Bath, UK; 8 CHU Sainte-Justine Azrieli Research Centre, Department of Psychiatry and Addictology, University of Montréal, Montréal, QC Canada; 9 Mila—Quebec AI Institute, Université de Montréal, Université de Montréal, Montréal, QC Canada

**Keywords:** Cognitive neuroscience, Human behaviour

## Abstract

We examine the neurobiology of intuition, a term often inconsistently defined in scientific literature. While researchers generally agree that intuition represents “an experienced-based process resulting in a spontaneous tendency toward a hunch or hypothesis,” we establish a firmer neurobiological foundation by framing intuition evolutionarily as a pathfinding mechanism emerging from the brain’s optimization of its relationship with the environment. Our review synthesizes empirical findings on intuition’s neurobiological basis, including relevant brain networks and their relationship to cognitive states like insight. We propose that unsolved problems dynamically alter attractor landscapes, guiding future intuitions. We investigate “opportunistic assimilation” through nonlinear neurodynamics and identify hippocampal sharp wave ripples as potential neural correlates of intuition, citing their role in creativity, choice, action planning, and abstract thinking. Finally, we explore intuition through two complementary perspectives: the free energy principle, which models brains as minimizing uncertainty through predictive hierarchical coding, and metastable coordination dynamics, describing the brain’s simultaneous tendencies toward regional cooperation and functional autonomy. Together, these principles provide a comprehensive neurodynamical account of intuition’s neurophenomenology.

## Abstract (web summary)

This Perspective article proposes a neurodynamical model of intuition as a fast, embodied pathfinding mechanism shaped by evolution. Drawing on the free-energy principle and metastability, it explores how the brain predicts, feels, and acts through dynamic coordination.

## Introduction

This is a paper about intuition. Over the years, researchers have used a series of definitions for this term, but Zanders, Olinger and Volz^1–3^ have argued that the consensus is “an experience-based process resulting in a spontaneous tendency toward a hunch or hypothesis.” As such, intuition often functions as informed judgment in the context of discovery, guiding individuals from tacit recognition of coherence to explicit insights, possibly highlighting a continuum rather than a dichotomy between nonconscious and conscious processes^2^. While the definition might only be “a general consensus,” it does capture an interesting phenomenological aspect of the experience: that it includes a subjective sense of knowledge (the “hunch” or “hypothesis” to which Zanders refers), as though facts have been gathered and a decision made—although the actual decision-making process remains hidden from view, both objectively and subjectively. This sense of knowledge also arises as a bodily sensation: a gut instinct or hunch^3^.

While intuition is a celebrated aspect of human experience, the lack of a rigorous, operationalized definition for the term (Abernathy and Hamm^4^, for example, identified at least twenty different definitions) stems from disagreements about the neurobiological and neurodynamical processes underlying intuition, and questions about varieties of intuition. Is intuition a singular experience, wherein the same structures and processes involved in, for example, Albert Einstein’s mathematical intuition, are also involved in William Butler Yeats’ poetic intuition?

Recent research has begun to address these issues, clarifying some of the neurobiological mechanisms of intuition, especially as they relate to emotion, reasoning, perception, insight, and decision making^1,5–7^. Yet, many open questions remain: How is intuition distinct from other cognitive processes, such as memory? Is there a neurobiological signature that correlates with intuition? What role do the dynamics of large-scale brain networks play in this process? Is there a neurophenomenology of intuition, and a unifying theory of brain function that explains both the phenomenology and the empirical data? The influence of emotional states on intuitive judgments is interesting, with positive moods enhancing and negative moods impairing the ability to intuitively make coherent judgments^8^. Lastly, in what way is intuition an embodied process, and what role does interoception play in this process? In this paper, we will address these questions from the perspectives of neuroscience, psychology, and philosophy to explore the neurodynamics of intuition and elucidate possible answers, testable hypotheses, and experimental models.

From a neuroscientific perspective, Zander’s definition spotlights three essential components of intuition. First, it suggests the involvement of nonconscious processes and, from an information-theoretic perspective, the transfer of information. It is worth mentioning that we consistently use the term information in this paper in a Shannonian sense, as an entropic reduction of uncertainty. Second, this information is brought quickly into conscious awareness. Third, a strong sense of agency and veracity accompanies the arrival of this information^1^. Thus, any comprehensive examination of intuition must explain the neurobiology and neurophenomenology of these three elements. In this paper, we intend to do just that.

Before diving into the details, it is helpful to discuss these ideas from a broader evolutionary perspective. Intuition, insight, and analysis are three processes that humans use to solve problems and make decisions. This raises a question: What kinds of decisions did these systems evolve to make? Here, we propose an answer. It is well-established that brains evolved to control movement^9,10^. Additionally, movement requires prediction, which Llinas and Roy^11^ termed the “prediction imperative,” stating that: “the intelligence component of motricity requires, for successful wheeling, a prediction imperative to approximate the consequences of impending motion.” Seth^12^, Buzsáki and Draguhn^13^, Buzsáki^14^, Freeman^15^, Kelso^16^, and Friston^17^ have all argued that broader mental states, including agency, cognition, and consciousness, derive from prediction. Thus, it is likely that all three cognitive processes—intuition, insight, and analysis—originated as ways to predict the consequences of impending motion. In this paper, we consider motricity and the predictive imperative as crucial to the enactive and embodied neurobiology of intuition.

To explore this concept further, we introduce the term “pathfinding.” In robotics and computer science, pathfinding commonly refers to the process an agent uses to find the most efficient or optimal path through an environment^18–20^. Typically, pathfinding algorithms process a sizable number of variables, spanning from spatial location to route selection to goal hierarchy assessment. Here, we aim to expand that definition to account for biological pathfinding necessities, including how an organism perceives and processes its environment, encodes spatial information, and tests environmental predictions via action, in what has been called the action-perception cycle^21,22^. In this sense, pathfinding is closely related to the use of affordances, landmarks, path integration, cognitive maps, social information, and movement through the action-perception cycle^23,24^. Additionally, we also use the term pathfinding to refer to the cognitive processes, both conscious and unconscious, that organisms use to navigate their environment in accordance with their goals, which includes intuition. We use the term pathfinding not merely as a metaphor borrowed from robotics, but as a conceptual bridge linking embodied movement, spatial inference, and nonconscious decision-making. To clarify its use, consider the following thought experiment: an organism faces a novel problem with no clear solution or precedent, such as a scientist encountering an unfamiliar dataset. There is no pre-learned script to follow, yet a directional “sense” emerges, guiding them toward the next step. This guidance cannot be reduced to rule-based analysis or associative memory recall alone. Instead, it resembles a form of intuitive navigation through a high-dimensional cognitive space, akin to pathfinding through an uncertain terrain. From a technical standpoint, this form of intuitive navigation can be approximated as paths of least action through a belief space—where, under the free energy principle, such paths are modeled as minimizing the integral of variational free energy over time, effectively optimizing posterior beliefs about the causes of sensory input^25^. We propose that such intuitive directional signals emerge from dynamic, embodied inferences over possible future states, thus justifying the term pathfinding as central to our hypothesis. Without it, we believe we lack a language for describing how intuition moves not just toward correctness, but toward coherence and action.

Functionally, then, we see intuition as an embodied, nonconscious pathfinding mechanism, that helps an organism decide where and how to move next—wherein, in cases like mathematical intuition or metaphorical intuition, we are considering the “solution” (that is, the answer to a math problem or the meaning of a metaphor) as a destination, the final step in a path. Mathematically, in terms of the Bayesian mechanics that attends the free energy principle, the notion of a path can be generalized to mean the path of least action; namely, the trajectory or narrative that minimizes the path integral of expected free energy that in turn minimizes expected surprise or uncertainty^25^. We note that while the proposed frameworks in this paper draw on the free energy principle and metastability^26,27^, we acknowledge that many real-world pathfinding problems faced by organisms—such as selecting among vast, complex alternatives—are computationally intractable (especially in a Bayesian sense, see discussion below). Classic problems like the Traveling Salesman^28^ and Knapsack^29^ problems offer useful analogues here, emphasizing the complexity of real-world decision-making^30,31^. However, rather than computing exact probabilities, we propose that biological systems employ neurodynamical heuristics that approximate solutions efficiently.

This means that the result of this search, the minimization of the path integral, can be considered in terms of “uncertainty” about the destination. With intuition, as Zhang^32^ has shown, the signal itself is binary: yes/no, go/no-go, approach/avoid. The signal is directional, providing the organism with pathfinding information. Yet, this same signal still leaves much uncertainty about the nature of the path itself. With insight, the signal becomes clearer and contains more external information about the path, but there is still uncertainty about the details. With analysis, uncertainty is reduced further, as every step in the path is now understood (for details about insight and analysis, see Kounios and Beeman^33^). In line with Walter Freeman’s argument about the “intentional arc” (see discussion below; Freeman^34^), which also suggests a tentative order to this process—with intuition evolving first, as a kind of proto-directional signal, and insight and analysis coming later, once organisms evolved the neural capabilities to process more complex information. This raises two critical details about intuition: one theoretical, the other speculative. Theoretically, as we explore below, intuition is about reducing uncertainty in a Bayesian sense of the term. More speculatively, intuition is to agency what insight and analysis are to consciousness, with “agency” being the term that evolutionary neurobiologists use to describe the flexible decision-making abilities found in almost all living organisms, from cells to human infants^35,36^. We will revisit these concepts as we move along.

To explicate these ideas, we examine what researchers have learned to date about the neurobiology of intuition. Next, we separate and examine three overlapping phenomena: intuition, insight, and analysis^32,33,37^, which extends our discussion from the neurobiology of intuition into its underlying neurodynamics. To expand further upon the neurodynamics, we consider the work of Colleen Seifert. In her psychological research into insight, Seifert^38^ built upon earlier work by Chase and Simon^39^, and proposed a distinct mechanism for storing important, yet unsolved problems. These problems serve as guides for future intuitions and insights. Here, we explore Seifert’s hypothesis from a systems neuroscience perspective, arguing that rather than being stored in anatomical structures, these unsolved problems form an “attractor landscape” that is subject to the laws of nonlinear dynamical systems, including the free energy principle and metastability (more on this below).

Building atop these foundations, we further propose hippocampal sharp wave ripples as neural correlates of intuition. Research shows that sharp wave ripples are involved in combining preexisting knowledge with recently acquired information to influence creativity, choice, action plan selection, and abstract thinking^40–42^. These short-lasting fast oscillatory patterns, which are the most synchronous neuronal-population firing pattern in the brain, occur during “off-line” states such as sleep, and also during waking states and are associated with compressed replay of spiking that occurred during waking experience^40,43,44^. However, this replay is often mixed with patterns that reflect the combination of previous experiences or “preplay” potential future choices. We will examine how sharp-wave ripples are involved in pathfinding and how the information they generate is processed by task-relevant neuronal structures and networks and then brought into awareness via interoceptive processes, the “gut instinct” or hunch (For more details, see Buzsáki^14,40^).

Finally, we attempt to answer a key question: Which modern theory of brain function best explains intuition? To accomplish this, we draw upon the two aforementioned theories: the free energy principle^45,46^ and metastability^16,26,47,48^, utilizing components from both to provide a neurodynamical account of the neurophenomenology of intuition. The free energy principle is based on the concepts of Bayesian updating and offers a modeling framework in which the brain is cast as relying on predictive hierarchical coding. It posits that brains, and potentially all living systems, use prediction to minimize uncertainty (as quantified by free energy) and resist disorder. This concept is equivalent to entropy, or expected surprise^49^ in the Shannonian sense. Essentially, all brains have beliefs about what causes particular incoming sensory information (priors, in Bayesian terms). These beliefs are continually updated via the action-perception cycle and the process known as “active inference”^50^, helping an organism optimize its internal representation of the external world and better navigate it. This is also why the free energy principle has been discussed as a modeling framework for understanding potential neurobiological mechanisms underlying creativity and insight^51,52^. Here, we extend this work to intuition. Now, although we frame intuition partly in terms of Shannonian information theory, we acknowledge that real-world environments are far too complex for organisms to acquire complete information about all relevant variables. In practice, intuitive judgments likely emerge from selective sampling of salient information, with intrinsic or epistemic value, rather than exhaustive information acquisition. This perspective aligns with ecological and embodied accounts of cognition, in which organisms optimize behavior under constraints, using limited but relevant information to motivate action (e.g., Schwartenbeck^53^).

Metastability^47^ plays a central role in cortical coordination dynamics. It refers to the simultaneous tendency for individual components of a system to couple together while also remaining autonomous. In cognitive neurodynamics, for example, different regions of the cortex, composed of distinct neuronal populations, can simultaneously coordinate their behavior to produce cognitive functions, while also expressing their own individual oscillatory behavior. This allows the brain to rapidly shift its functionality to make sense of the external world. Metastability is critical for cognition^54^, especially in situations that require sensitivity to both novelty and possibilities for action^55^, which are two processes involved in intuitive pathfinding. We argue that metastability can help explain intuition in several ways. First, the metastable brain might balance various cognitive processes dynamically, allowing intuitive judgments to emerge without overt analysis. Moreover, as we explore below, intuition relates to past experiences and pattern recognition. In the metastable brain, large-scale brain networks are quickly accessed and interconnected, allowing for the subjective felt-sense of a solution before slower processes take over. Second, for intuition to occur, the brain might rapidly and flexibly integrate disparate pieces of information from various sources, crucially, in a non-linear fashion. Third, intuitive judgments may be transient, i.e., arising and fading suddenly. This is aligned with metastability as a description of transient tendencies—that is, those that exist between fully stable states. As a final thought, both free energy minimization and metastability have adaptive value, meaning they have been selected for in an evolutionary sense^55^.

The free energy principle and metastable coordination dynamics could be conceptually viewed as two sides of the same coin, although further empirical validation is needed to support this alignment. This follows from the fact that the free energy principle casts internal (e.g., neuronal) dynamics as a gradient flow on variational free energy, thereby furnishing paths of least action. Variational free energy provides an upper bound on surprise. Therefore, minimizing variational free energy minimizes surprise and uncertainty. This can also be expressed as maximizing the evidence for generative models of the lived world, or self-evidencing^56^. Crucially, minimizing variational free energy maximizes the entropy of posterior beliefs, in the spirit of the maximum entropy principle, to which the free energy principle is dual^57^. This may help explain features of metastability and related neuronal dynamics, such as criticality^17^. Heuristically, this means that to self-evidence is to keep an open mind (cf., Occam’s principle), through coordinated flows on variational free energy landscapes that—by construction—feature metastability.

## An overview of intuition neuroscience

**Fig. 1:**
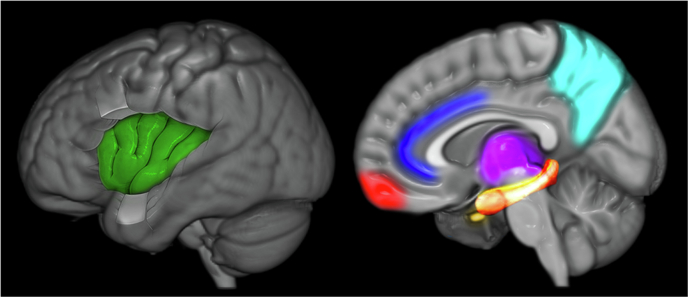
The neural networks of intuition. The figure illustrates the distributed neural architecture underlying intuitive cognition, characterized by metastable coordinated activity across multiple brain regions. Key structures include: orbitofrontal cortex (red) integrating sensory-emotional inputs for coherence assessment; anterior cingulate cortex (blue) regulating cognitive transitions; anterior insula (green) processing interoceptive signals; basal ganglia (purple) facilitating implicit pattern recognition; hippocampal-entorhinal complex (orange) mediating experience replay via sharp-wave ripples; amygdala (yellow) incorporating emotional valence in risk evaluation; and precuneus (teal) supporting self-referential processing. Together, these regions form a dynamic network that oscillates between integration and segregation states, with intuition emerging as a product of neurodynamical coordination that minimizes uncertainty through rapid action-path prediction and selection.

While research into the neural correlates of intuition is sparse, especially when measured against other aspects of decision-making, there have been attempts to examine its phenomenology and neurobiology. One important avenue of research has been a series of imaging studies of intuitive judgments of coherence^2,6,7,58–61^. In these studies, participants made spontaneous judgments about “coherence,” determining whether a certain object or sound or word shared patterns, structure or meaning with others in a given set, or what vision scientists sometimes call “gist perception”^62,63^. They found that a judgment of coherence correlated with early activation in the orbitofrontal cortex (OFC), an area that receives input from all sensory modalities and is crucial for emotionally-driven decision-making. Furthermore, this activity also correlated to the phenomenological experience of intuition, and could be separated from the characteristics of the stimulus, the requirements of the task, and the participant’s explicit recognition of the object, sound or word^60,61,64,65^. Research has identified several brain regions consistently involved in intuitive processes (summarized in Fig. 1). For instance, the orbitofrontal cortex plays a key role in emotionally-driven decision-making and coherence judgments, while regions such as the anterior insula and hippocampus contribute significantly to the embodied and predictive aspects of intuitive judgments (see discussion below).

For instance, in their 2006 fMRI work on intuitive judgments of visual coherence, Volz and von Cramer^6^ presented participants with pixelated drawings of everyday objects. These were rated as either coherent (those dots look like a violin) or as incoherent (those dots don’t look like anything). Drawings judged as “intuitively coherent” correlated with activity in the medial OFC. This finding was in line with earlier work by Bar^66^ that suggests the OFC provides an initial guess at coherence, and that this guess serves a gating function, thereby limiting the number of object representations that need to be considered. Importantly, Volz and von Cramer do not see this as an example of top-down processing, stating: “The gist information is realized on the basis of the observer’s implicit knowledge rather than being consciously extracted on the basis on the observer’s explicit knowledge”^6^. (See below for further discussion).

Horr^60^ harnessed the superior temporal resolution of magnetoencephalography (MEG) to show that the OFC is an early integrator of incomplete stimulus information. Using a semantic coherence task, they found that OFC activation increases as soon as stimuli produce a phenomenological, subjective feeling of coherence, before any activation occurs in temporal object recognition areas. Moreover, this activity appears even earlier for coherent stimuli than incoherent stimuli. They also found that when words were presented sequentially over time, rather than all at once, there was a dramatic change in neuronal activation patterns. These changes in patterns involved a shift from the left OFC to the right OFC, and suggest that there may be a second system designed to handle information that is accumulated gradually.

In an fMRI study of a semantic coherence task, Zander^61^ extended this research in a number of important ways. In this study, participants were shown three words and asked if they were related or unrelated to a fourth word shown after a slight delay. Researchers found that intuition emerges gradually, through a process of “spreading activation,” wherein judgments result from “the activation of remotely linked semantic concepts that do not yet lead to full conscious retrieval”^60^. These nonconscious perceptions are similar to priming-based judgments in their ability to influence subsequent behavior, and resemble, the authors state: “the definition of implicit memory^3^.” The fact that researchers have consistently linked intuition to implicit memory implicates the basal ganglia as another relevant region of interest, as this structure has been repeatedly linked to that process^67–69^. This idea receives further confirmation from Wan^70,71^, who found that activation in the caudate nucleus, a part of the basal ganglia, correlated with the development of skills-expertise and intuitions about that expertise. Additionally, Remmers^72^ discovered that the accuracy of intuition was negatively influenced by anxiety, which also corresponds to anxiety’s impact on implicit memory, or what is sometimes termed “implicit memory bias”^73^.

Finally, Mega^74^ used fMRI to investigate intuitive versus deliberate judgments during an emotional facial recognition task. They speculate that their results argue against popular dual system accounts, which hold that intuitive and deliberate judgments are supported by two distinct cognitive processing mechanisms^75^. Importantly, this study represents one of the first attempts to investigate the underlying neural architecture of these two systems, wherein the dual systems model would predict distinct neural activation patterns. In this study, participants were asked to judge the authenticity of an emotional facial expression. One group was asked to go with their initial “gut” feeling, while the other was asked to take their time and be deliberate. In general, the researchers found similar neural activation patterns in both tasks, supporting a unified decision-making model wherein both intuitive and deliberate judgments rely on similar neural processes. However, some distinctions were also found. In the intuitive condition, there was activation of left OFC, precuneus, intraparietal lobula, and bilateral anterior insula. The bilateral anterior insula is intricately connected with the OFC, and is known to be involved with interoception^76^ and the accuracy of interoceptive feelings^77^. They believe that these connections may instantiate the feeling of intuition. In other words, these somewhat distinctive activation patterns reflect two of the defining features of intuition: nonconscious processing and phenomenological signaling.

## An overview of insight research: a systems neuroscience perspective

In addition to these coherence studies, researchers have also examined the similarities and differences between intuition and insight. In this work—and in line with the aforementioned research—intuition is seen as coherence, or the ability to create nonconscious links between information. Insight, meanwhile, is the process by which the sudden comprehension and resolution of a situation arises into consciousness^78^. Thus, one major distinction between intuition and insight is the transition of information into conscious awareness^6^, with the former being implicit and the latter explicit. Zander^1^ consider the explicit part of this process an integrative or second stage of intuition. Zhang^32^ take this further, seeing intuition and insight as distinct processes, with key neuronal, behavioral, and phenomenological differences. During intuition, for example, unconsciously activated neural information guides the subject toward a binary (yes/no) decision, with little concern for the results of that decision. This is contrasted with insight, wherein the concern is with the nature of the solution, not with a binary judgment about its coherence. Interestingly, intuition and insight differ significantly in problem-solving, with intuition often guiding noninsight problem-solving through a gradual recognition of coherence, whereas insight involves a sudden realization and explicit understanding of the solution^79^.

Another difference between intuition and insight is how each process starts. Drawing upon the entropic brain hypothesis and the free energy principle, Carhartt-Harris and Friston^52^ clarified the mechanics of insight, arguing that “insight often occurs as part of a process, the initial phase of which involves an intention or plan, e.g., to discover something new.” When viewed through the lens of predictive hierarchical coding, this urge to discover something new causes the brain to relax its confidence in previous assumptions or, in Bayesian terms, “high-level priors.” According to Friston^46^, the relaxation of high-level priors manifests behaviorally as open-minded curiosity, or what’s sometimes called the “beginner’s mind”^80^. With insight, the relaxation of high-level priors reflects conscious decision-making and an ongoing search process—a plan to discover something new. With intuition, we propose that the relaxation of high-level priors is triggered (or cued) by novel information in the environment or as the result of internal neuronal events. In either case, this information is both noticed and processed nonconsciously.

A related distinction between intuition and insight involves the final step in the process, or the nature of the “solution signal” itself. With insight, the solution can be expressed verbally, marking the point at which a solution becomes linguistically expressible. From an information-processing perspective, this transition into language can be seen as a significant reduction of uncertainty, in line with the free energy principle^46^. In Bayesian terms, this may correspond to model selection and reduction: pruning less likely hypotheses in favor of a parsimonious explanation^52^, which has been correlated with goal-directed behavior^50^. In contrast, intuition also involves a reduction of uncertainty, but it does so below the threshold of linguistic expression. The intuitive signal, such as a hunch or gut feeling, may reflect an implicit or embodied registration of coherence that remains inaccessible to conscious verbalization^32^. Thus, from a Bayesian standpoint, intuition could be framed as a sub-personal form of model updating that has yet to consolidate into explicit knowledge.

On this view, intuition could be regarded as a sub-personal exploration of plausible hypotheses through short-term plasticity and synaptic replay^81^, until an “Aha! moment” is reached and a suitably simple explanation for the sensory evidence at hand is encountered (see Friston^51^ for a numerical illustration). At this point, uncertainty is resolved, and the confidence or precision ascribed to various (Bayesian) beliefs increases in a way that can be recognized, leading to insight and (propositional) beliefs that can be reported. Interestingly, the increase in the precision of beliefs about path plans has been associated empirically with phasic dopaminergic discharges^53,82,83^, which speak to the affective valence of insight^84,85^ and thereby the possible implications for interoceptive inference and realization of ‘gut feelings’^86–89^.

A final group of studies has attempted to tease apart the neural underpinnings of insight versus analysis^33,90^. These studies have utilized fMRI, EEG, and the Remote Association Test (RAT), which is similar, yet different from the semantic coherence tasks used in intuition research. Similarly, both tasks present a triad of words to participants. In semantic coherence, a fourth word is shown that coheres or does not cohere (in meaning, structure, or pattern) with the already presented triad. In the Remote Association Test, a triad of words is shown to participants, and the task is to find a fourth word that can modify all three. For example, “apple, grass, sand” is the triad, with “crab” being the insightful solution. In general, insight is correlated with greater activity in the right hemisphere than in the left, as the former has been linked to coarser semantic coding and is more likely to find broader and less obvious solutions^33^. Interestingly, during the “preparation for insight” phase, these studies also showed activation in the bilateral temporal cortices, the anterior cingulate cortex, and the hippocampus. While the role of the hippocampus will be explored below, these results raise questions concerning the OFC. If intuition is a gradual process with insight being the end of that process, why was there no OFC activity during the remote association test? Likewise, in the semantic coherence task, it is unclear why no activity was observed in the anterior cingulate cortex, the very region previously associated with conflicting solutions or strategies^91,92^. Lastly, right before the arrival of an insight solution, researchers also found a burst of activity in the alpha band in the occipital cortex that was immediately followed by a burst of gamma activity over the right temporal lobe. They argue that this pre-insight alpha burst reflects “transient sensory gating that reduces noise from distracting inputs to facilitate retrieval of the weakly and unconsciously activated solutions represented in the right temporal lobe”^76,90^. Thus, these results may be partially indicative of the nature of the task itself—RAT versus semantic coherence—but they also highlight the neuronal differences between intuition, insight, and analysis. Moreover, the role of processing fluency and affect in intuitive judgments has been studied, showing that increased fluency and positive affect can significantly influence the perceived coherence of word triads, independent of their actual semantic relationship^93^. Last, research indicates that intuitive judgments of semantic coherence can be made swiftly and reliably, even under tight response protocols, suggesting that such judgments rely heavily on nonconscious processes^64^.

Taken together, we propose that these studies provide initial evidence for a neurodynamical approach to intuition. Their findings are consistent with multiple theoretical frameworks, including the free energy principle, which may contribute to explaining intuitive judgments. Their findings also suggest roles for several brain regions in the intuitive process, including the orbitofrontal cortex, anterior cingulate cortex, anterior insula, basal ganglia, hippocampus, and entorhinal cortex.

## The neurodynamics of opportunistic assimilation

In the foregoing sections, we reviewed prior neurobiological research into intuition, insight, and analysis. Methodologically, these phenomena were tested in the laboratory with a series of contrived problems, wherein participants were consciously aware that there was a task to complete in a short time span. However useful these experiments are, they can be markedly different from the real world situations that produce intuition and insight. In the real world, individuals are not always aware that there is a problem to solve, where to hunt for solutions, nor what kind of solution would be most useful. Additionally, when solutions do arrive, many show up after a considerable delay—that is, a long time after the original problem was encountered.

Seifert^38^ explored a scenario where an individual encounters a critical problem, which they fail to solve, yet their brain recognizes the importance of the problem and stores all the details. Later, if the individual comes across a piece of information that can complete the puzzle, it is rapidly recognized and utilized to create a solution. A hypothetical example might be useful: A neuroscientist is frustrated by a problem in the lab and decides to take a break and walk down a tree-lined street. Along the way, she passes a majestic oak. Five minutes later, she has an intuition: a gut instinct that tells her to stop walking and look at the trees. She doesn’t understand this feeling—its meaning remains opaque. Yet the sensation is powerful enough that she stops walking to stare at the trees. Some minutes later, while still staring at the trees, the scientist experiences an Aha! Moment wherein she realizes the tree’s branches resemble the structure of a dendrite, and her observation of this branching structure solves a problem she has been working on in the lab—an idea she then rushes back to the lab to test. In other words, awareness of the problem remains nonconscious until the solution itself arrives. This raises another essential distinction between intuition and insight. Insight is characterized by feelings of unexpectedness, elation, satisfaction, and conscious awareness—the phenomenological characteristics of an Aha! moment^33^. Intuition, meanwhile, is an embodied sensation marked by uncertainty about its origin and meaning.

The above scenario is an example of Wallas’s^94^ classic four-stage “cycle of creativity”: preparation, incubation, illumination, and verification. Poser^95^ called this the “prepared-mind perspective.” Seifert^38^ attempted to formalize this model, proposing that the mind cycles through a series of information processing stages that begin with *preparation*, or the explicit consideration of a problem. This is followed by *incubation*, which entails setting the problem aside and thinking about something else for a while. Next are the third and fourth stages: *illumination* and *verification*—that is, the arrival of the insight itself, followed by its validation. More importantly, the authors propose that, during the preparation stage, when the mind fails to find a solution to the problem, these failures get stored in long-term memory where they can help steer the individual toward a later insight—a phenomenon they term *opportunistic assimilation*. In opportunistic assimilation, the memory of an unsolved problem guides future information processing by nonconsciously preparing the brain to recognize potential solutions. This process, it is worth mentioning, is similar to how the brain approaches unmet goals^42^, and appears to work much like priming^96^, except over considerably longer time scales.

To understand how opportunistic assimilation functions over longer time scales, it’s helpful to consider the process from the perspective of cognitive neurodynamics. The field of neurodynamics emerged partially from work in coordination dynamics^97,98^, which examines coordinated behavior at every level of scale, from the coordination of neuronal activity that leads to an intuition, to the coordination of bees in a swarm, to the coordination of stars in a galaxy. As framed by Kelso^16^ and Kelso and Engstrom^99^, the field asks the question: “How do individual elements of any system at any level of description come together to form coherent patterns of behavior, along with the form that such coherence takes?” These coherent or—to use the more common term—“stable” patterns are referred to as “attractors.” When an individual has an intuition and/or an insight, this is an example of a stable attractor, or a solution to a problem that is a temporary point of stasis in an otherwise dynamic environment. When viewed through the lens of the free energy principle, this solution can be modeled as a path of least action toward a free energy minimum, corresponding to a metastable set of neuronal states.

One of the central discoveries of coordination dynamics^47,54^ is metastability, a basic property of any system of coupled oscillators, including neuronal oscillators. As mentioned, a system is metastable when its dynamics tend toward a stable attractor (coordination pattern) but are never entirely trapped by that attractor. Metastability allows a system to remain poised on the edge of criticality, where it has remarkable information processing capabilities^100^ and maximum cognitive and behavioral flexibility, without descending into disorder^101,102^. Intuition and insight, in order to yield useful information for an organism, may require this level of flexibility. In this sense, metastability can be considered a kind of “enabling mechanism” for both the prepared mind hypothesis and the process of opportunistic assimilation.

Importantly, in metastable cortical coordination dynamics, information can be “meaningful”—in that it helps the brain coordinate behavior—but without being consciously recognized as so^48^. This is what happens during opportunistic assimilation. The storage of the unsolved problem in long-term memory changes an attractor landscape, allowing the brain to move closer to the edge of criticality and, ultimately, a single attractor—that is, the solution. Thus, rather than being stored in distinct anatomical structures, we propose that these indices of failures to solve problems form a multistable attractor landscape^98^ that may reflect heightened uncertainty (i.e., excess of free energy), which the system tends to resolve through unconscious processes. Thus, when any potential solution to either a part or the whole of this unsolved problem is discovered—or in situations high in novelty, where a solution is most likely to be discovered—we see a dynamical shift in the brain, and this marks the beginning of the process we call intuition. And, in the fullness of time, insight.

This shift, we propose, represents a relaxation of high-level priors, a move toward increasing entropy in the hopes of obtaining “optimal grip”—a term coined by philosopher Merleau-Ponty^103^. Normally, optimal grip refers to an organism’s attempt to understand and then maximize the potential benefits of all of the available affordances in an environment—with *affordances* referring to J.J. Gibson’s description of objects as “opportunities for action”^104^. In their work on skilled intentionality and Gibsonian neuroscience, Bruineberg and Rietveld (2014) draw upon a minimal interpretation of the free energy principle to position cognitive neurodynamics within the larger dynamics of the self-organizing brain-body-environment system, which, they posit, allows for a formalization of optimal grip. Applying this to intuition, we go one step further; While Merleau-Ponty’s use of word “environment” refers to the external, physical world^103,105^, here we argue that there is also a form of “cognitive optimal grip,” where internally-generated thoughts, especially partial solutions to important unsolved problems, can also be included in the search. The resulting cognitive optimal grip, which, in the terms of traditional intuition research, could be termed “coherence”, is then broadcast to the rest of the system via interoceptive mechanisms and, possibly, for validation purposes (see discussion below), corollary discharges^34^.

From the perspective of coordination dynamics, this “coordination” can be read as generalized synchrony or synchronization of chaos (inherent in nonlinear neurodynamics). This ensures maximum mutual information (and information transfer) across distributed neuronal systems, with a deep hierarchical structure. Generalized synchrony of this sort emerges as neuronal dynamics are drawn to synchronization manifolds that constitute the attracting sets with a low variational free energy^106,107^. See Friston and Frith^108^ for numerical studies of generalized synchrony and the emergence of optimal grip in the context of hermeneutics and communication.

Yet, to obtain an optimal grip, one first needs to relax one’s grip. This implies a shift from the quasi-optimal grip that characterizes the attractor landscape of unsolved problems to a state of increased entropy—the relaxation of high-level priors—in order to introduce novelty and the possibility of new solutions (or the restoration of actual, no longer “quasi,” optimal grip). This is in line with Carhartt-Harris and Friston’s approach to insight^52^, but applied to intuition. In short, when an unsolved problem’s attractor landscape precludes the resolution of uncertainty, the brain moves towards the edge of criticality, in order to obtain an optimal grip, and reduce free energy^51,53^.

We note, however, that cognitive optimal grip does not rely on an exhaustive analysis of all environmental variables. Instead, it emerges through interaction with structured regularities— intrinsically motivated (i.e., epistemic) affordances, heuristics or priors, and salient cues—within high-dimensional and dynamically shifting environments. In this view, intuitive behavior reflects an embodied engagement with both environmental and internal constraints, enabling adaptive responses in computationally challenging, real-world contexts.

Taken together, both the free energy principle and metastable coordination dynamics provide explanations of how the brain uses prediction, uncertainty, surprise, and flexibility to produce intuition. But questions remain. Does the brain index critical unsolved problems, such as survival or long-term unmet goals, as failures? After all, ongoing survival is not only the most critical problem an organism needs to solve; the problem itself cannot be solved—thus, are existential threats considered unsolved problems? Can this help explain why gut instincts—don’t walk down that alley—happen so quickly? Do failure indices get established in a single swoop (like our frustrated neuroscientist who goes for a walk), or can they be established by open-loop problems or existential threats that cannot be solved?

## The neurobiology and neurodynamics of intuitive pathfinding

To begin to answer these questions, we expand upon a previously discussed idea, which is the evolutionary argument that intuition is a pathfinding mechanism, helping an organism determine where and how to move next. In this sense, pathfinding is closely related to using affordances, landmarks, path integration, cognitive maps, social information, and moving through the action-perception cycle^23,24^. Additionally, we also use pathfinding to refer to the cognitive processes, both conscious and unconscious, that organisms use to navigate their environment in accordance with their goals, and this includes intuition. We attempt to do this by introducing empirical and theoretical evidence showing that intuition correlates to activity in the hippocampal-entorhinal system, which are the two main neurological structures associated with pathfinding and many of the subcomponents that make path-finding possible: space, time, intentionality or agency, and action-planning^14,34^.

Specifically, the hippocampus contains place cells, which specialize in tracking discrete locations, while the entorhinal cortex contains grid cells, which specialize in tracking possible paths between these locations. Importantly, this same pathfinding architecture forms the basis of episodic and semantic memory, wherein events and facts are processed as discrete locations, while all the associations between them are processed as paths. Importantly, the hippocampus and entorhinal cortex not only store locations, events, and facts from the past (memory); they also use those locations, events, and facts to chart possible paths forward (action planning). Buzsáki and Llinas state: “Navigation and memory are deeply connected. Analogous to map- and path-based navigation, there are two forms of hippocampal system–dependent memories: memorized facts (or semantic memory) and one’s personal experiences (episodic memory). To reexperience egocentric episodes, we project ourselves back in space and time (episodic recall), whereas traveling into the imagined future represents planning (prediction).”^109^ This aligns with findings on the preselection effect in short-term motor memory, where planned movements are more accurately reproduced than passive or constrained ones, likely due to corollary discharge signals that predict the sensory consequences of intended actions^110^.

Both of these ideas are further bolstered by the work of Walter Freeman^34^, who noted that intentional action—or what could be considered the source of “causal agency”—begins with future goals and the actions required to reach those goals^34^. This is the aforementioned “intentional arc.” Additionally, Kelso and Fuchs^36^ proposed a computational model of Rovee-Collier’s mobile conjugate reinforcement of a prelinguistic baby interacting with the world^36^. In their model, the sense of self emerges from the coupling between the baby and its environment, and they demonstrated that a sense of self emerges; a sense of agency is experienced, i.e., the Aha! moment of “making something happen in the world.” Closely related to this, Freeman’s work also shows that action demands prediction, and characterizes emotion as a kind of prediction, or what he called the “stretching forth of intentionality” and the “anticipation of future action.”

In this framework, intuition is a predictive emotion representing the expected outcome of a particular path of action. This emotion may be considered a “summary signal,” combining predictions about multiple action-planning issues. For example, the risk-level of an impending action and the energetic requirements of that action do not arise into conscious awareness as separate signals, and are instead summarized by a single predictive phenomenological sensation—an intuition. Equally critical, Freeman also argued that this signal originates in the hippocampus. As he states: “The global interaction of the motor, sensory and associational areas create a spatiotemporal pattern that is conceived to express the present brain state^111^. Emergence of the pattern requires participation of the allocortex, in particular the hippocampus. The present state evolves into a prediction of a future state that contains within it a plan of action to achieve that state: “the corollary discharge signals to the parietal cortex, resulting in covert ‘actions,’ would allow the brain to evaluate the potential consequences of future plans. Without any overt behavior, the mirror and corollary discharge systems can then inform the brain’s owner that she is the *agent* of this covert activity.”^14^ (emphasis ours). Thus, whenever an individual with an unsolved problem encounters a potential solution, that solution can be tested against the predictions captured in the attractor landscape and represented by those corollary discharges, and this mechanism might be involved in the formation of a sense of agency.

Given the preponderance of evidence pointing toward the hippocampus as a crucial structure in intuition as pathfinding, we can now extend this argument to hippocampal sharp wave ripples—a neurodynamical process—which we propose as another neural correlate of intuition. Research shows that sharp wave ripples are a pathfinding mechanism that combines preexisting knowledge with recently acquired information to influence creativity, decision-making, action-plan selection, and abstract thinking^40–44^. Sharp wave ripples have been observed in every mammalian species examined thus far, including humans. They are comprised of a “sharp wave”— that is, a negative polarization in the hippocampal CA1 layers—and the “ripple”: a short-lived fast oscillation (140–200 Hz) of the local field potential. These waves are the most synchronous neuronal-population firing pattern in the brain. They occur during “offline” states such as sleep, but also during waking states. In both cases, they facilitate the replay of spatial and temporal waking neuronal sequences, but in a short, compressed manner. This compressed information can be replayed forward (in the order the experience occurred) or backward^112^, and then transferred to other brain regions that support learning, memory consolidation, and action planning. Essentially, sharp wave ripples are a subconscious mechanism that allows an organism to explore options. They search memory for stored sequences of past events to predict future outcomes. They also compress information from the past and predictions to aid problem-solving and augment path-planning. In their empirical work on memory in rats, Fernando Ruiz^42^ states, “sharp wave ripples can be conceived as an internalized vicarious trial-and-error process that flexibly ‘imagine’ real or fictive alternatives to select an optimal path or construct novel inferences without the need for movement-based exploration.” Meanwhile, the failure to replay the appropriate trajectory sequence before a choice is made can predict behavioral errors.

Additionally, sharp wave ripples help unite and explain a number of unresolved findings about intuition. For example, Norman^113^ used a visual learning paradigm to discover that both the successful encoding of information and the successful recall of that information were preceded by higher incidences of ripples, arguing that “hippocampal ripples may thus boost recollections during episodic memory retrieval.” This finding may explicate the phenomenological sensation of intuitive accuracy (that a gut instinct is correct) and uncertainty (that the origin of this gut instinct is unclear) that accompanies the conscious perception of an intuition. If the brain uses ripples to increase recall precision, it makes sense that this extra neuronal processing step would be represented in phenomenological experience as a signal that the information is accurate. Yet, since ripples are a nonconscious process, we would not be able to detect why we feel this information is accurate (or, in neurodynamic terms, why uncertainty has been reduced—only that it has). In a related finding, Cox^114,115^ found that there are also sharp wave ripples in the amygdala, which coordinate with the hippocampus during NREM sleep. This may explain the powerful emotional quality of intuition and how the signal itself contains both pathfinding information (normally localized in the hippocampus-entorhinal cortex complex^14^) and risk-assessment information (normally localized in the amygdala^116^). Finally, the notion that sharp wave ripples are key neurophysiological components of intuition is in line with the literature on naturalistic decision making, which defines intuition as decision making based on “situational pattern recognition”^117^. In this framework, intuition requires a comparison between a current situational pattern and situational patterns stored in memory from previous experiences, or what is termed the “recognition-primed decision model^118^ ”. In short, a metastable attractor landscape that is created by unsolved problems and activated by sharp wave ripples is a putative mechanism for situational pattern recognition—an intuition.

**Fig. 2:**
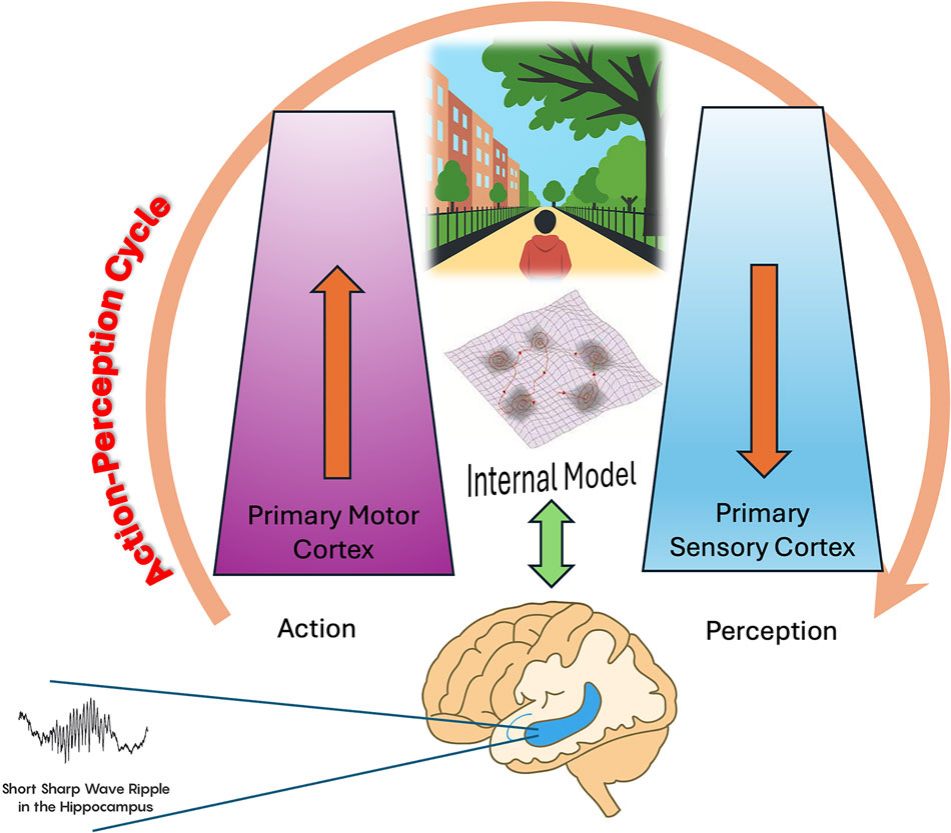
A neurodynamical framework of intuition within the action-perception cycle. The schematic depicts how intuitive cognition emerges through the dynamic integration of sensory and motor hierarchies. The central dynamic attractor landscape represents the internal model that integrates sensory input and motor output to facilitate intuitive judgments and decision-making. The sharp wave ripple inlet symbolizes hippocampal contributions, combining past experiences with present stimuli to shape predictions and pathfinding. The framework is grounded in the interaction with the external environment, highlighting the embodied and predictive nature of intuition.

Finally, intuition can be visualized within a neurodynamical framework (see Fig. 2), where sensory inputs and motor outputs interact dynamically within the perception-action cycle. The sharp wave ripples illustrated in Fig. 2 highlight the crucial hippocampal contribution, integrating past and present information to inform intuitive judgments and facilitate effective pathfinding.

## The neurodynamics of intuition: a theoretical outlook

In this section, we return to metastability and the free energy principle, exploring both as powerful theoretical frameworks for intuition. Metastability, as described above, refers to the co-existence of stability and flexibility in neurodynamics, whereby brain regions simultaneously integrate and segregate local and global oscillatory activity^47,98,119^. More specifically, the brain is highly dynamic, with different regions constantly interacting and forming transient networks. Metastable coordination dynamics allow for the rapid integration and processing of information across these networks^120^. Kelso^48^ demonstrated that these dynamics underlie slower conscious thought processes, such as decision-making by analysis. We hypothesize that metastability also underpins the rapid decision-making required by intuition. By facilitating the formation of flexible networks, metastability allows for the seamless integration of information from both conscious and nonconscious processes, leveraging implicit knowledge structures and past experiences to create intuitive judgments.

More formally, intuition requires the activation of relevant associations—the attractor landscape. To make use of that landscape, both pattern recognition and pattern prediction are necessary^121^. Because intuitive judgments can arise quickly, all three processes must occur at high speeds. Through metastable dynamics, the brain can operate on the edge of criticality, allowing rapid access to its repository of learned patterns, and the ability to use these patterns to generate real-time predictions about current circumstances^122,123^. Recently, Hancock^124^ provided a comprehensive review of the thirty-plus-year history of the concept of metastability, emphasizing its significance in understanding brain function and its role in optimal brain function. Metastable coordination dynamics, originating in dynamical systems theory, are characterized by a balance between integrated brain activity and bursts of desynchronization for specialized functions. It has applications in cognitive performance, aging, meditation, and the treatment of various neurological conditions. Challenges exist, however, particularly in measuring metastability; they are primarily due to the process’s mathematical complexity and the need for heuristic markers. Of course, further research is required. Additionally, metastability allows the brain to swiftly generate these coordinated responses without the need for explicit reasoning or analytical thinking—an idea in alignment, we argue, with intuition as rapid nonconscious information processing.

Meanwhile, the free energy principle is a theoretical framework rooted in Bayesian inference and prediction. The principle states that the brain continuously updates its internal models to minimize prediction errors (i.e., surprise, quantified as “variational free energy”) in order to optimize its relation to, and interaction with, the external world^46,125^. It accomplishes this by attempting to reduce the uncertainty, or prediction error, between external sensory information and internally generated models. Fundamentally, the free energy principle is a statement about a situated agent’s interaction with its environment, whether those interactions are perceived consciously or not.

Importantly, as the entire body-brain system subserves the agent’s interactions, “embodied cognition” also plays a crucial role in the free energy principle^126^. Embodied cognition refers to the fact that cognitive processes are integrated with an agent’s physical body and its interactions with its environment, and that this combination shapes cognition. This is a causal relationship. Many features of cognition are deeply dependent on the physical body—meaning the body plays a causal role in cognitive processing^127–129^. The free energy principle builds on this idea, stating that an agent continuously optimizes the relationship between its physical body and the environment via “active inference,” or the brain’s continuous generation of predictions and the action adjustments needed to minimize prediction errors^108^. In the context of intuition, active inference involves the brain rapidly (via metastability) generating predictions based on implicit knowledge and past experiences, and using those predictions to guide decision-making without conscious deliberation. It is worth noting that this description of intuition is in line with how the brain utilizes active inference to process affordances, or what might be described as proto-intuitive pathfinding^55^.

Active inference is an application of the free energy principle to sentient behavior that foregrounds embodied action upon the world, in the spirit of active sensing and learning. A key aspect of active inference—that speaks to a universal kind of pathfinding—is that agents entail a generative model that includes the consequences of their own action^14^. This immediately casts overt and covert action in terms of planning as inference^92,130,131^. In active inference, the likelihood that an agent will commit to a given plan rests on the free energy expected if the organism were to follow that plan. The expected free energy can be decomposed into expected information gain and expected cost, where cost is just the prior surprise associated with uncharacteristic outcomes. The key point here is that to evaluate the expected free energy of a path into the future, it is necessary to rehearse that path; speaking to phenomena such as (p)re-play activity and the putative importance of sharp wave ripples: e.g., Stoianov^132^. The expected information gain is clearly an important aspect of intuition, which lends any plan an epistemic affordance^53,133–135^.

We note here a distinction between intuition and inference. We argue that intuition can be understood as a form of active inference operating at sub-personal, nonconscious levels. It reflects the rapid deployment of prior expectations encoded in hierarchical generative models, triggered by environmental cues. While fast and feedforward, it still contributes to uncertainty minimization, though often in a coarse manner rather than through deliberative model updating. Thus, the difference between intuition and inference, in the language of the free energy principle, is not categorical but a matter of degree and temporal resolution. Intuition reflects fast, heuristic inference operating at lower levels of the hierarchy, typically without top-down cognitive access or reflective goal modulation. In contrast, deliberate inference engages slower, higher-level loops that allow for the flexible revision of priors and explicit planning. Still, both are enacted through the same underlying imperative: to reduce expected free energy and optimize agent-environment coupling.

Embodied cognition emphasizes the fact that the brain’s internal models are built upon sensorimotor contingencies, or the law-like regularities in the relationship between bodily action and the resulting sensory feedback^136,137^. As the brain moves through the action-perception cycle, sensorimotor contingencies form the basis for predictive models^138^. By integrating sensory information with motor signals and bodily states, the brain predicts the consequences of the organism’s actions, in line with the predictive imperative discussed in the previous section. If intuition is rooted in motricity, then sensorimotor contingencies play a crucial role in the process^137^. This means intuitive pathfinding is a form of nonconscious embodied cognition, with active inference as an underlying mechanism, which is also in line with how the brain processes affordances^55,139^.

The role of interoception in intuition is also underpinned by embodied cognition and the free energy principle. It is well known that interoceptive signals, such as heart rate and respiratory rate, and visceral sensations like gut movements, impact cognitive processes^140,141^. By providing valuable information about internal states, these signals also contribute to the brain’s predictive imperative, helping to optimize the body-brain-environment system by reducing uncertainty. Kandasamy^142^, for example, found that the interoceptive abilities of London stock traders (their ability to listen to gut instincts) predicted their relative profitability and how long they survived in financial markets. Another line of evidence for this comes from Allen, Levy, Parr, and Friston^25^, who used a computational model based on emerging empirical data to show that an agent’s beat-to-beat heart rhythm contributes to their affective perceptual beliefs. Then, by “lesioning” the model, the researchers showed that perception changes and belief models get updated (more formally, uncertainty) as posterior entropy increases.

Finally, the free energy principle states that all internally generated models are hierarchical^25^. This matters here, as intuition demands accurate predictions made in short timeframes and based on implicit knowledge and prior experiences. Hierarchical models allow for the rapid integration of information across different levels of abstraction, providing a rich context for generating the accurate predictions that underlie intuitive judgments. This also helps explain why Kahneman and Klein^143^ found that having expertise about a subject improves the accuracy of intuitive judgment. Expertise involves building better hierarchical models, which minimize prediction error.

In summary, both metastability and the free energy principle offer useful theoretical frameworks for explaining how intuition might operate in the brain. Of course, these two approaches should be considered alongside other theoretical perspectives as the field continues to develop. Aligned with the strong currents in modern systems neuroscience, we argue that self-organized dynamics play an essential role in the neural basis of intuition. Metastability allows for the rapid integration of information and the activation of relevant associative networks. The free energy principle emphasizes the generation of accurate predictions, the refinement of internal models, the reduction of uncertainty, and active inference. While we note that the intimate relationship between metastability, brain state shifts, the free energy principle, and intuition is only conceptual at this point, we emphasize the preliminary explanatory power of this theorizing when applied to the phenomenon.

## Alternative interpretations

While we have proposed a neurodynamical framework that positions intuition as an embodied, predictive, and metastable pathfinding mechanism, operating under active inference, it is important to acknowledge that alternative interpretations remain viable. Traditional dual-process theories, for instance, distinguish between fast, automatic (System 1) and slow, deliberative (System 2) processes, often assigning intuition to the former^144,145^. Our account does not reject this dichotomy but instead reframes it within a continuous dynamical systems perspective, wherein both intuition and analysis emerge from shared underlying principles—specifically, hierarchical predictive coding and the resolution of uncertainty across time scales. In this view, the difference lies not in categorical systems but in the speed, accessibility, and neurodynamical profile of the cognitive state.

Moreover, some may interpret intuition as the byproduct of associative learning mechanisms, particularly in domains where expertise has been developed. From this perspective, intuitive judgments may not require the dynamics of attractor landscapes or hippocampal sharp wave ripples, but rather emerge from conditioned patterns of recognition and response. While our framework accommodates this by proposing that such heuristics are encoded within the same generative models that support active inference, we recognize the need for empirical work that can disentangle these explanations. Whether hippocampal replay uniquely contributes to intuitive decisions or reflects general memory consolidation and planning remains an open question. Our goal here is to provide a generative, testable framework that can integrate these perspectives, rather than to exclude them.

## Conclusion

In this paper, we have attempted to establish a neurobiological and neurodynamical framework for intuition. Our work suggests that metastable coordination dynamics and the free energy principle offer complementary theoretical frameworks for approaching the phenomenon of intuitive judgments. We also propose hippocampal sharp wave ripples as a putative neurophysiological mechanism for this process, with the orbitofrontal cortex, anterior cingulate cortex, anterior insula, basal ganglia, amygdala, hippocampus, and entorhinal cortex all appearing to play key roles. Furthermore, the work emphasizes intuitive pathfinding as nonconscious embodied cognition—that is, a decision-making process rooted in motricity and agency. We have also argued that intuition, insight, and analysis are underpinned by distinct neurodynamical processes, which should include changes in cortical functional connectivity. Particularly, although intuition and insight do share some overlap, they are phenomenologically different, and some aspects of insight, such as conscious retrieval, Aha! Moments and opportunistic assimilation may be explained from the perspectives of computational neuroscience and nonlinear dynamics. (See Table 1 for a succinct overview of these various concepts and theories.)

**Table 1:** Core concepts in the neurodynamical account of intuition

Concept/theory	Definition/description	Role in intuition
Intuition (core definition)	Nonconscious, embodied, fast-acting process that guides decision-making via directional “hunches”	Serves as an early pathfinding mechanism rooted in motricity, prediction, and agency
Free energy principle	Brains minimize prediction error (“surprise”) through hierarchical Bayesian inference.	Explains how intuition reduces uncertainty nonconsciously via predictive modeling and active inference
Metastability	Dynamic regime balancing stability and flexibility in brain network coordination.	Enables rapid, flexible switching between cognitive states, supporting the emergence of intuitive judgments
Sharp wave ripples (SWRs)	Synchronous hippocampal events replaying past experiences in compressed time	Proposed physiological basis for intuition; supports internal simulation and memory-guided pathfinding
Attractor landscapes	Dynamic topography of possible brain states shaped by past experiences and goals	Intuitions emerge as transitions between attractors, influenced by stored unsolved problems.
Pathfinding	Cognitive and neural processes of selecting viable paths toward goals	Central framing concept for intuition as an adaptive mechanism for navigating uncertain environments
Opportunistic assimilation	Reactivation of stored unsolved problems when new, relevant information is encountered	Explains delayed intuitions emerging from dynamic reorganization of previously encoded experiences
Intuition–insight–analysis continuum	A sequence from fast, uncertain feelings to conscious realization and step-by-step reasoning	Positions intuition as early-stage signal; insight as realization; analysis as explicit understanding
Interoception and embodied cognition	Bodily signals (e.g., gut, heart, breath) informing cognition and perception	Provides the somatic “felt sense” of intuition; links body-state to emerging awareness.
Agency and motricity	Evolutionary roots of cognition in movement and goal-directed behavior	Frames intuition as a sensorimotor-based predictive tool guiding adaptive action in the world

While our primary focus has been on the neurodynamical mechanisms underpinning intuition, it is important to acknowledge that intuitive judgments are not always accurate. The process of intuition, like all predictive processes, is inherently probabilistic and therefore susceptible to error. In certain contexts, especially under conditions of ambiguity or novelty, intuitions may yield maladaptive or suboptimal inferences. This is evident in reversal learning paradigms, where prior associations must be rapidly unlearned, and intuitive responses can persist despite environmental change^50,143,146^. Nonetheless, such outcomes do not disqualify the process as intuitive. Rather, they reflect the broader adaptive function of intuition as a fast, resource-efficient mode of navigating uncertainty. This process trades precision for speed and operates below the threshold of conscious deliberation. As such, even erroneous intuitions remain part of the same underlying pathfinding mechanism. Overall, we hope our proposal contributes to the ongoing effort to develop a neurobiological account of intuition and offers testable hypotheses for future investigation.
